# Influence of motivation, self-efficacy and situational factors on the teaching quality of clinical educators

**DOI:** 10.1186/s12909-017-0923-2

**Published:** 2017-05-08

**Authors:** Christoph Dybowski, Susanne Sehner, Sigrid Harendza

**Affiliations:** 1 Department of Internal Medicine, University Medical Center Hamburg-Eppendorf, Martinistr. 52, Hamburg, 20246 Germany; 2 Institute for Biometrics and Epidemiology, University Medical Center Hamburg-Eppendorf, Martinistr. 52, Hamburg, 20246 Germany

**Keywords:** Teaching quality, Clinical teaching, Clinical teacher, Undergraduate education, Motivation, Physician, Self-determination theory, Self-efficacy, Student evaluation of teaching

## Abstract

**Background:**

Being exposed to good teachers has been shown to enhance students’ knowledge and their clinical performance, but little is known about the underlying psychological mechanisms that provide the basis for being an excellent medical teacher. Self-Determination Theory (SDT) postulates that more self-regulated types of motivation are associated with higher performance. Social Cognitive Theory (SCT) focuses on self-efficacy that has been shown to be positively associated with performance. To investigate the influences of different types of teaching motivation, teaching self-efficacy, and teachers’ perceptions of students’ skills, competencies and motivation on teaching quality.

**Methods:**

Before the winter semester 2014, physicians involved in bedside teaching in internal medicine at the University Medical Center Hamburg-Eppendorf completed a questionnaire with sociodemographic items and instruments measuring different dimensions of teaching motivation as well as teaching self-efficacy. During the semester, physicians rated their perceptions of the participating students who rated the teaching quality after each lesson. We performed a random intercept mixed-effects linear regression with students’ ratings of teaching quality as the dependent variable and students’ general interest in a subject as covariate. We explored potential associations between teachers’ dispositions and their perceptions of students’ competencies in a mixed-effects random intercept logistic regression.

**Results:**

94 lessons given by 55 teachers with 500 student ratings were analyzed. Neither teaching motivation nor teaching self-efficacy were directly associated with students’ rating of teaching quality. Teachers’ perceptions of students’ competencies and students’ general interest in the lesson’s subject were positively associated with students’ rating of teaching quality. Physicians’ perceptions of their students’ competencies were significantly positively predicted by their teaching self-efficacy.

**Conclusions:**

Teaching quality might profit from teachers who are self-efficacious and able to detect their students’ competencies. Students’ general interest in a lesson’s subject needs to be taken into account when they are asked to evaluate teaching quality.

**Electronic supplementary material:**

The online version of this article (doi:10.1186/s12909-017-0923-2) contains supplementary material, which is available to authorized users.

## Background

Medical education research provides evidence that clinical teachers influence students’ performance: being exposed to good teachers is associated with better clinical performance and greater medical knowledge of students [1–3]. Three main categories of characteristics of good clinical teachers have been identified: 1) knowledge, competencies, and skills as a physician, 2) enthusiasm for medicine and teaching, and 3) general positive human characteristics such as communication skills and respect for others as reflected in a supportive learning environment [4]. However, little is known about the underlying psychological structures that provide the basis for being a good clinical teacher.

One of these underlying structures might be motivation, which can be defined as “those psychological processes involved with the arousal, direction, intensity, and persistence of voluntary actions that are goal directed” [5]. Among many motivational theories, predominantly Self-Determination Theory (SDT) [6] has influenced educational research in the context of work and organizational psychology in the last two decades. SDT proposes a multidimensional view of motivation and distinguishes between three major types of motivation depending on the level of involved autonomy or self-determination: autonomous motivation (comprising intrinsic motivation and identified regulation), controlled motivation (comprising external regulation and introjected regulation), and amotivation [6]. SDT postulates that more self-determined types of motivation are associated with higher effort in actions at which the motivation is targeted, and empirical findings show that they are associated with greater commitment and better performance regarding these actions [7]. Furthermore, studies from non-medical settings demonstrate that teachers’ autonomous teaching motivation can foster autonomous learning motivation in their students [8–10], and students’ autonomous learning motivation can positively affect academic performance [11]. The potential of SDT for medical education has been acknowledged by Ten Cate et al., who also advise to consider teacher motivation in educational research [12].

Another psychological mechanism with potential relevance for high quality teaching is perceived self-efficacy, which constitutes a central construct of Social Cognitive Theory (SCT) and which can be defined as the extent to which a person believes to be able to successfully complete an action [13, 14]. According to SCT, self-efficacy beliefs affect both motivation and performance [13]. Meta-analyses provide clear evidence for the positive relationship between self-efficacy beliefs and work performance [15, 16]. In non-medical settings, the impact of teacher self-efficacy on students’ academic achievement has already been demonstrated [17–19]. Regarding teachers, higher teaching-self-efficacy was associated with a more persistent behavior [20] and with striving for improved didactic methods [21].

Recent studies in medical education research suggest that, when investigating the effect of teachers on students, the effects of students on teachers also have to be considered in order to control for potential confounding. Two qualitative studies imply that teachers’ perceptions of their students within the teaching context might play an important role on their situational motivation [22, 23]. They identified two main categories of characteristics of “good students” from the perspective of educators: skills/competencies and conduct [22, 23]. Teachers prefer enthusiastic, motivated, proactive, respectful, and disciplined students [22, 23]. Based on these findings, we assume that teacher’ perceptions of student behavior within the teaching may also influence teaching behavior and quality.

Therefore, we hypothesize that autonomous types of teaching motivation are associated with higher teaching quality than controlled types of teaching motivation. We also assume that teaching amotivation is negatively associated with teaching quality. Furthermore, we assume that teaching self-efficacy is positively associated with teaching quality. Finally, we assume that teachers’ positive perceptions of students’ behaviors are positively associated with teaching quality.

## Methods

### Study design

We conducted a prospective observational study with clinical teachers from the Department of Internal Medicine at the University Medical Center Hamburg-Eppendorf. Internal Medicine comprised the subspecialties cardiology, endocrinology, gastroenterology, infectious diseases, nephrology, oncology and pneumology. All teachers were physicians (residents or consultants) employed at our University Medical Center with their main task being patient care. The learners were students form our traditional curriculum (semester 5 to 8 of 12) and from our vertically integrated curriculum (semester 5 of 12), which were both offered at the University Medical Center Hamburg-Eppendorf at the time of data collection. We chose bedside teaching (BST) as the type of lesson for our study as it constitutes a large part of both undergraduate medical curricula and physician-patient interaction represents an important part of students’ future work. Students rotate between different wards every week and encounter different teachers. Teachers are selected by their respective departments and need to be at least first year residents. Every single BST encounter comprises 45 min (in both curricula), in which three to eight students visit one or two patients and practice history-taking as well as physical examination under the supervision of one teacher. Selection of patients occurs by the individual clinical teacher based on learning objectives for the respective clinical discipline [24].

Each of the 85 physicians who had been scheduled for at least one bedside teaching lesson during the winter semester 2014/15 (October 2014 to February 2015) received a paper-and-pencil questionnaire 3 weeks before the beginning of the semester which contained socio-demographic items, an instrument measuring different dimensions of teaching motivation, and an instrument measuring teaching-related self-efficacy (see below). During the semester after each lesson, physicians received a questionnaire with five short items regarding situational factors, which might have influenced the lesson (see below). At the same time, students filled out a questionnaire rating several aspects of teaching quality with respect to the particular BST lesson (see below). In order to counteract potential loss of motivation for filling out the questionnaires, we raffled 10 book vouchers at a value of 25 Euro each at the end of the semester.

### Instruments

#### Teaching motivation

Teaching motivation was measured using the Physicians’ Teaching Motivation Questionnaire (PTMQ), which is a validated multidimensional self-assessment instrument based on SDT containing the subscales ‘intrinsic teaching motivation’, ‘identified teaching motivation’, ‘career teaching motivation’, ‘introjected teaching motivation’, ‘external teaching motivation’ and ‘teaching amotivation’ [25]. In a validation study, the factorial validity of the instrument, its concurrent criterion validity as well as its incremental validity over global work motivation were confirmed [25].

#### Teaching self-efficacy (TSE)

In order to assess TSE, we used the Physician Teaching Self-Efficacy Questionnaire (PTSQ) [26]. This validated scale consists of 16 items that represent typical critical situations regularly faced by medical teachers such as time strain, problems with patients and patient selection, interruptions of the lesson, short-term allocation of teachers to lessons, or unmotivated students [23, 24, 26, 27]. A five-point Likert-scale of agreement was used for the rating of these items.

#### Situational variables

After each lesson, physicians rated their perceptions concerning three situational student variables, which were identified as important in a previous study: students’ motivation and engagement, the appropriateness of students’ previous skills and knowledge with respect to the lessons content as well as students’ punctuality and other indicators of respect [23]. As studies suggest that workload can influence teaching quality [28, 29], we also included one item each for a) physicians’ perceptions of having had enough time to prepare the lesson and b) for having felt stressed before the lesson due to other work tasks as potentially confounding variables. All situational variables were rated on a five-point Likert scale of agreement. The original and translated items are provided in Additional file 1.

#### Teaching quality

For the student ratings of teaching quality, we used 13 items from three validated questionnaires: the Maastricht Clinical Teaching Questionnaire (MCTQ) [30], a validated German questionnaire for the evaluation of seminars in universities (FESEM) [31] and a German questionnaire for the generic evaluation of teaching (HILVE II) [32]. Furthermore, we complemented these existing items with self-constructed items. This approach was necessary because none of these instruments was fully applicable to assess the quality of teaching within the context of BST. The means of the selected items represent four indicators of teaching quality: learning climate, behavior towards patients, didactics, and motivation and enthusiasm. As ceiling effects are known in student evaluations of teaching [33], we tried to prevent this by providing a nine-point Likert scale of agreement and by giving short instructions on how to use the scale with each distribution of the questionnaire. Items were formulated positively (e.g. “She/he gave me constructive and useful feedback”) and rated on a nine point Likert scale of agreement ranging from 1 (“does not apply at all”) until 9 (“applies without any restrictions”). The individual items, their means and the internal consistencies of the subscales are provided in Additional file 2.

#### Socio-demographic characteristics and confounders

We collected the following data to be included as potential confounders in our statistical model: teachers’ age and sex, teaching experience in years, having participated in our medical centers’ teacher training program and students’ sex. Furthermore, students’ general interest was included as it has been identified as a potential source of bias in medical students’ ratings of teaching [34].

### Statistical analyses

Missing values were replaced using the expectation-maximization algorithm in SPSS. First, we performed a confirmatory factor analysis (CFA) to examine the structural fit of the student ratings of teaching quality to the data.

In order to examine the influences of teacher dispositions and situational variables on teaching quality, we performed a mixed-effects linear regression. This model accounts for the hierarchical data structure involving teachers who had several lessons and the same students rating different teachers due to rotations within the semester. For the resulting three cluster levels (teacher, student, lesson) we included random intercepts for each with the following modeling approach: teaching lessons were modeled as nested within single teachers, which were modeled as crossed between student ratings. As our primary dependent outcome variable, the four subcategories of the student ratings were modeled as repeated measures of teaching quality. Additionally, we included a variable that identifies the four subcategories to estimate potential mean differences between them. We assumed that resulting residuals were identical and independently distributed. The five motivation scores, the teaching self-efficacy score as well as the five situation variables were simultaneously modeled as predictors of teaching quality. Furthermore, to avoid potential confounding, student characteristics (gender, general interests in topic) and teacher characteristics (age, gender, teaching experience, participation in teacher training) were included in the model.

In a second exploratory step, we examined the influence of the physicians’ dispositions on those situational variables that significantly predict student ratings of teaching quality (in this case teachers’ perceptions of their students’ prior knowledge and competencies) as dichotomous variable with a mixed effects logistic regression. Because each teacher assessed several lessons only once, the unit “teacher” was modeled as a random intercept and teachers’ motivation and self-efficacy scores as fixed effects. As before, the teacher characteristics were included as confounders. Nominal *p*-values are reported without correction for multiplicity. Two-sided *p*-values <0.05 were considered as significant. The factorial structure of teaching quality as rated by the students was analyzed using IBM AMOS 22, all other analyses were conducted with StataCorp Stata 14. The Ethics Committee of the Hamburg Chamber of Physicians confirmed the innocuousness of this study and its congruence with the Declaration of Helsinki. No questionnaires contained names; instead, anonymous identification codes were used to match questionnaires by the same persons.

## Results

### Sample

75 teachers (88.2%) returned their questionnaires measuring teaching motivation and teaching self-efficacy. Data from 123 BST-lessons were collected. 13 lessons could not be analyzed due to missing teacher questionnaires measuring motivation and/or teaching self-efficacy, further 12 because of missing questionnaires with teachers’ perceptions of a lesson, 2 because no student ratings were available and 2 because no information regarding teaching experience was given. Five student ratings had to be excluded from analysis due to missing students’ identification codes. This resulted in 94 lessons held by 55 different teachers suitable for analysis (Table 1), in which 237 different students filled out 500 questionnaires after the lessons, resulting in an average amount of 5.3 student ratings per lesson (range 1 to 9). 88 lessons (93.6%) were rated by three or more students.

**Table 1:** Sociodemographic characteristics of the physician sample (*n* = 55)

	M ± SD / %
Age (years)	34.9 ± 6.9
Sex	
Male	61.8%
Female	38.2%
Teaching experience (years)	6.2 ± 6.3
Participation in teacher training	
Yes	29.1%
No	70.9%
Occupational position	
Resident	69.1%
Consultant	9.1%
Attending physician	20.0%
Other	1.8%

Among the motivational categories, identified teaching motivation was most pronounced, followed by intrinsic teaching motivation (Table 2). Among the situational variables, teachers’ perceptions of students’ respect were most pronounced, while the impression of having had enough time to prepare the lesson was least pronounced. Our assumed factorial structure of one superordinate factor indicating general teaching quality and comprising the four subcategories learning climate, behavior towards patients, didactics as well as motivation and enthusiasm showed acceptable to good fit after deleting one item for learning climate (RMSEA = .078, CFI = .972, TLI = .956, SRMR = .032). Learning climate received the best student ratings (M = 8.4, SD = 1.1), while didactics received the worst (M = 7.9, SD = 1.3). As students’ ratings of teaching quality displayed a strong ceiling effect, the data were transformed by calculating the logarithmized values.

**Table 2:** Means of teaching quality, teaching motivation, teaching self-efficacy and situational variables

	N	M ± SD
Outcome: Teaching quality^a^		
Didactics	499	7.9 ± 1.3
Learning climate	500	8.4 ± 1.1
Motivation and enthusiasm	500	8.1 ± 1.3
Behavior towards patients	488	8.1 ± 1.2
Motivational categories^b^	55 for all	
Intrinsic TM		2.7 ± 0.9
Identified TM		3.2 ± 0.7
Introjected TM		0.7 ± 0.8
External TM		1.5 ± 1.0
T Amotivation		0.9 ± 0.9
Teaching self-efficacy^b^	55	2.5 ± 0.5
Situational variables^b^	94 for all	
Enough time to prepare lesson		2.1 ± 1.2
Stress before lesson		2.2 ± 1.1
Perceived students’ motivation		3.1 ± 0.7
Perceived students’ competences		2.9 ± 0.8
Perceived students’ respect		3.6 ± 0.6

*TM* Teaching Motivation, *T* Teaching

^a^scale from 1 to 9 with 9 = best rating

^b^scale from 0 to 4, higher values representing stronger manifestation

### Predictors of teaching quality

Analyses indicated that there were no linear relationships between the situational variables as perceived by the teachers and students’ ratings of teaching quality. Therefore, the situational variables were treated as categorical variables in the following analyses.

As for the confounders, students’ general interest in the subject of a lesson was significantly positively associated with students’ ratings of teaching quality (factor: 1.07; 95%-CI 1.05–1.08; *p* < 0.001; Table 3). As for physician’s personal dispositions, no type of teaching motivation nor teaching self-efficacy were significantly associated with students’ ratings of teaching quality. As for the situational variables, the physicians’ perceptions of their students’ competencies were significantly associated with students’ ratings of teaching quality (*p* = .004). The categories that express a stronger agreement than the category “hardly applies” did not differ significantly among each other, but each category showed significantly higher ratings in comparison to “hardly applies”.

In a next step, we investigated associations between the teachers’ perceptions of their students’ competencies and teachers’ dispositions in a mixed effects logistic regression (Table 4). Physicians’ perceptions of their students’ competencies were significantly positively predicted by their teaching self-efficacy (OR = 24.66; 95%-CI 1.45–418.18; *p* = .026). With a smaller effect size, teaching amotivation was also significantly positively associated with physicians’ perceptions of their students’ competencies (OR = 5.61; 95%-CI 1.12–28.17; *p* = .036).

**Table 3:** Predictors of teaching quality

Predictors	Unstandardized adjusted est. Parameter (Factor)	95%-CI		*p*	*p* global
Physician demographics					
Age	0.97	0.95	1.00	.055	
Sex (ref.: “female”)	0.98	0.86	1.13	.807	
Teaching experience (years)	1.02	0.99	1.05	.168	
Participation in teacher training (ref.: “no”)	1.06	0.89	1.26	.542	
Physicians’ teaching motivation (PTMQ)					
Intrinsic	0.90	0.78	1.04	.141	
Identified	0.97	0.81	1.15	.697	
Introjected	0.99	0.89	1.11	.890	
External	0.90	0.79	1.00	.053	
Amotivation	1.01	0.88	1.15	.896	
Physicians’ teaching self-efficacy (PTSQ)	1.13	0.91	1.39	.254	
Situational variables as perceived by the physicians					
Stress due to other tasks	-	-	-	-	.648
Enough time for preparing the lesson	-	-	-	-	.778
Students’ motivation	-	-	-	-	.129
Students’ prior knowledge and skills (ref.: “hardly applies”)	-	-	-	-	*.004*
- “partly applies”	1.69	1.24	2.30	*.001*	
- “rather applies”	1.62	1.14	2.29	*.007*	
- “completely applies”	1.72	1.22	2.44	*.002*	
Students’ respectful behavior					.420
Student variables					
Sex (ref.: “female”))	0.94	0.87	1.01	.111	
General interests in topic	1.07	1.05	1.08	*<.001*

**Table 4:** Logistic regression of physicians’ perceptions of their students’ competences on physicians’ dispositions

Physicians’ dispositions	OR	95%C		p
Demographics				
Age	1.17	0.85	1.61	.334
Sex (ref. “female”)	0.57	0.14	2.34	.439
Teaching experience (years)	0.80	0.53	1.21	.297
Participation in teacher training (ref. “no”)	0.37	0.07	1.85	.225
Teaching motivation (PTMQ)				
Intrinsic	0.92	0.24	3.56	.903
Identified	2.19	0.43	11.16	.345
Introjected	0.27	0.07	1.00	.050
External	1.66	0.47	5.79	.428
Amotivation	5.61	1.12	28.17	*.036*
Teaching self-efficacy	24.66	1.45	418.18	*.026*

*OR* Odd’s Ratio

The unit “physician” was modeled as a random intercept and physicians’ motivation and self-efficacy scores as fixed effects.

## Discussion

In contrast to the predictions of SDT and SCT, we found no direct impact of teaching motivation or self-efficacy on teaching quality. At the same time, student ratings of teaching quality were very high. This might indicate that, at least for the lessons we investigated, autonomous motivation or self-efficacy are not necessary to achieve high ratings for teaching quality during BST. A reason for this might be the relatively strong standardization of BST lessons in the departments we investigated, in which predefined standards for the learning goals and the execution of the lessons exist. Furthermore, it is possible that teaching motivation and self-efficacy exert an effect on other variables outside lessons that we did not measure, such as participation in the organization of teaching, and voluntarily offering to undertake more teaching lessons [25]. However, we found a positive association between ratings of teaching quality and teachers’ perceptions of students’ previous knowledge and skills. Perception of adequate knowledge and skills might result in teachers’ stronger involvement, leading to higher teaching quality [23]. A reason could be that teachers who rate their students’ competencies highly might have assessed their students’ prior knowledge and skills and adapted their teaching strategies accordingly, leading to higher student ratings. Activating prior knowledge has been found to be an important cognitive didactic approach for effective learning in education in general [35] and in medical education in particular [36]. On the other hand, studies suggest a positive association between grading leniency and student evaluations [37, 38]. In our study, the teaching physicians did not give grades, but it cannot be excluded that generally less strict and demanding teachers were given better student ratings.

Moreover, we found that teachers’ situational perceptions of their students’ knowledge and skills were predicted by teachers’ teaching self-efficacy. This might be due to some physicians’ ability and readiness to focus on strengths instead of weaknesses, applying this focus on their own capabilities as well as on their students’ and could be explained by the so-called psychological process of projection. This process has been demonstrated in studies from social psychology especially for members of the same group with which one identifies [39]. Teacher trainings could not only enhance physicians’ ability to detect their students’ competencies, but also their teaching self-efficacy. Furthermore, with a lower effect size, teachers’ situational perceptions of their students’ knowledge and skills were positively predicted by teaching amotivation. It is possible that teachers who are less motivated are also less demanding. However, this effect could also have been caused by less motivated teachers who did not fill out the questionnaires thoroughly and carefully.

Included as a confounder variable, students’ general interest in the subject of a specific lesson showed a positive association with ratings of teaching quality. This is consistent with the finding that prior interest in a certain topic influences student evaluations in higher education [34, 40]. While this effect is seen as a potential bias by most authors, others have questioned the causality of the association and suggest that frequent exposure to good teaching raises interest in a subject [41]. However, due to the design of our curriculum where students spend only short rotations in the different departments of internal medicine, it is unlikely that prior good teaching of a specific subject has influenced their interest in this particular subject in our study.

A strength of our study lies in focusing on an important clinical teaching format, BST, which enabled us to assess specific situational factors. On the other hand, due to the shortness of the BST lessons, students’ time spent with an individual teacher might have been too limited for teaching motivation and self-efficacy to unfold their impact effectively. A lack of enough time to experience a certain teacher might also have led to less differentiated student ratings as reflected by their low variance. Furthermore, the weekly evaluation of their teachers could have resulted in a loss of students’ motivation to fill out the questionnaires carefully. Therefore, an underestimation of the strengths of the association cannot be excluded. On the other hand, the rating of different teachers by the same students allowed us to statistically correct for students’ individual response biases. Our assessment of teaching quality might constitute a limitation to the interpretation of our results. First, the assessment of teaching quality by means of student ratings is not without controversy in the literature. While there is evidence that student ratings correlate with expert ratings, several potential sources of bias have been revealed as well [34, 42–45]. On the other hand, additional raters can increase the risk of reactivity [46], which occurs when observed individuals change their behavior or performance. Therefore, a strength of our study lies in the simultaneous consideration of basic dispositions and situational variables within a naturalistic setting. However, in future studies, more objective types of assessments might be employed. A second potential weakness of our assessment of teaching quality might be that we did not use a validated instrument. However, as we used categories of teaching quality that are described to be important in the research on good clinical teachers and partially adopted items from well-validated questionnaires, we believe that content and face validity can be assumed. In the confirmatory factor analysis of our scale for the assessment of teaching quality, the multilevel structure has not been accounted for, which could have resulted in a distortion of the results of the confirmatory factor analysis. Furthermore, in this study, the actual learning effects of the students have not been assessed. It might be possible that, while teachers’ motivations and self-efficacy do not affect student ratings, possibly due to rating bias, they might affect actual learning progress. Another potential weakness is that we did not assess students’ prior experience with BST. It can be assumed that students with more experience can differentiate stronger and therefore estimate the quality of teaching more precisely. Therefore, this potential moderator variable should be assessed in future studies.

Our results imply that teaching quality might benefit from training teachers in the ability to detect their students’ competencies and from enhancing physicians’ teaching self-efficacy. As postulated by Bandura and confirmed in various studies, the main sources of self-efficacy constitute mastery experiences, positive vicarious experiences, verbal persuasion and the subjective interpretation of physiological and affective states during an action [13, 14]. Teacher trainings should be based on these principles to effectively enhance physicians’ teaching self-efficacy. However, our results do not imply that teaching motivation is generally irrelevant to teaching quality. Our findings might be restricted to the special type of lessons we investigated and to our choices of assessment. Therefore, different types of lessons should be investigated in future studies as well as implications of teaching motivation apart from the actual teaching, e.g. the readiness to organize lessons, involvement in curriculum development and others. Furthermore, as autonomous types of motivation have been found to be associated with the well-being of employees, the teaching motivation of clinical teachers should also be considered from this perspective [6]. In addition, assessments of students’ learning progress, e.g. with OSCEs, might be are more reliable criterion for teaching quality than student evaluations of teaching quality and could help to further clarify the relationships between teaching motivation, teaching self-efficacy and teaching quality in clinical teaching.

## Conclusions

In conclusion, our findings indicate that neither teaching motivation nor teaching self-efficacy have a direct impact on teaching quality within our setting of BST. However, clinical teachers’ perception of students’ competencies are associated with higher ratings of teaching quality and are predicted by teachers’ self-efficacy. Furthermore, students’ general interest in a lessons’ topic seems to constitute a bias on student evaluations of teaching.

## Additional files

Additional file 1: Questionnaire for the situational variables as perceived by the physicians. English and original German items for measuring physicians’ perceptions of the situational variables. (DOCX 24 kb)

Additional file 2: Questionnaire for the student ratings of teaching quality. Scales and translated items of the questionnaire for the student ratings of teaching quality including means and standard deviations, Cronbach’s alphas and factor loadings in the confirmatory factor analysis. (DOCX 23 kb)

## Abbreviations

BST: Bedside teaching
CFA: Confirmatory factor analysis
PTMQ: Physicians’ Teaching Motivation Questionnaire
SCT: Social Cognitive Theory
SDT: Self-Determination Theory
TSE: Teaching self-efficacy
