# Supplementary material for: Influence of motivation, self-efficacy and situational factors on the teaching quality of clinical educators
Source: BMC Med Educ. 2017 May 8;17:84. doi: 10.1186/s12909-017-0923-2 (PMC5423026; doi:10.1186/s12909-017-0923-2)
Supplement: Supplementary file 1 — Questionnaire for the situational variables as perceived by the physicians. English and original German items for measuring physicians’ perceptions of the situational variables. (DOCX 24 kb) [file 12909_2017_923_MOESM1_ESM.docx]

**Additional file 2: Questionnaire for the situational variables as perceived by the physicians**

|  | | Does not apply at all | Does mostly not apply | Applies partially | Applies largely | Applies fully |
| --- | --- | --- | --- | --- | --- | --- |
| 1 | I had enough time to prepare the lesson I just taught. (German original: Ich hatte genügend Zeit, um den zurückliegenden Unterricht vorzubereiten.) | 🞎_0_ | 🞎_1_ | 🞎_2_ | 🞎_3_ | 🞎_4_ |
| 2 | At the beginning of the lesson, I was stressed out due to my other work obligations.  (German original: Ich war zu Unterrichtsbeginn durch meine anderen Arbeitsaufgaben gestresst.) | 🞎_0_ | 🞎_1_ | 🞎_2_ | 🞎_3_ | 🞎_4_ |
| 3 | The students in the lesson I just taught appeared to me to be motivated and involved.  (German original: Die Studierenden der zurückliegenden Unterrichtseinheit machten einen motivierten und engagierten Eindruck auf mich.) | 🞎_0_ | 🞎_1_ | 🞎_2_ | 🞎_3_ | 🞎_4_ |
| 4 | The students appeared to me as competent as I expected them to be in this phase of their undergraduate medical studies.  (German original: Die Studierenden wirkten so kompetent auf mich, wie ich es für diesen Studienabschnitt als angemessen empfinde.) | 🞎_0_ | 🞎_1_ | 🞎_2_ | 🞎_3_ | 🞎_4_ |
| 5 | The students were arrived on time and also obeyed other rules of mutual respect.  (German original: Die Studierenden waren pünktlich und hielten auch sonstige Regeln des gegenseitigen Respekts ein.) | 🞎_0_ | 🞎_1_ | 🞎_2_ | 🞎_3_ | 🞎_4_ |
