# Supplementary material for: Influence of motivation, self-efficacy and situational factors on the teaching quality of clinical educators
Source: BMC Med Educ. 2017 May 8;17:84. doi: 10.1186/s12909-017-0923-2 (PMC5423026; doi:10.1186/s12909-017-0923-2)
Supplement: Supplementary file 2 — Questionnaire for the student ratings of teaching quality. Scales and translated items of the questionnaire for the student ratings of teaching quality including means and standard deviations, Cronbach’s alphas and factor loadings in the confirmatory factor analysis. (DOCX 23 kb) [file 12909_2017_923_MOESM2_ESM.docx]

**Additional file 1: Questionnaire for the student ratings of teaching quality**

|  | **Scales and items (origin in brackets)** | **Mean (SD)** | **Cronbach’s α** | **Factor loadings^3^** |
| --- | --- | --- | --- | --- |
|  | **Learning climate** | – ^2^ | **.84**^2^ | **.85** |
| 1 | The teacher encouraged us to ask questions. (self)^1^ | 8.2 (1.3) |  | .26 |
| 2 | The teacher created a safe learning environment. (MCTQ) | 8.2 (1.3) |  | .92 |
| 3 | The teacher behaved in a friendly and respectful way towards the students. (FESEM) | 8.5 (1.0) |  | .82 |
|  | **Behavior towards patients** | – ^2^ | **.89** | **.92** |
| 4 | The teacher showed interest in the patient as a person. (self) | 8.2 (1.2) |  | .85 |
| 5 | The teacher served as a role model for the kind of doctor I would like to become. (MCTQ) | 7.9 (1.4) |  | .93 |
| 6 | The teacher treated the patient respectfully. (self) | 8.3 (1.2) |  | .86 |
|  | **Didactics** | – ^2^ | **.88** | **.87** |
| 7 | The teacher adjusted his/her teaching activities to my level of experience. (MCTQ) | 7.9 (1.4) |  | .78 |
| 8 | The teacher was supportive when I had trouble with a task. (MCTQ) | 8.1 (1.3) |  | .88 |
| 9 | The teacher gave us very useful and constructive feedback. (MCTQ, modified) | 7.7 (1.6) |  | .86 |
| 10 | The teacher tried to integrate all students into the lesson.  (self) | 8.0 (1.4) |  | .76 |
|  | **Motivation and enthusiasm** | – ^2^ | **.93** | .**90** |
| 11 | The teacher gave me a very motivated and involved impression. (self) | 8.2 (1.3) |  | .94 |
| 12 | I think the teacher really enjoyed the lesson. (self) | 8.0 (1.5) |  | .92 |
| 13 | I think our learning progress was truly important to the teacher. (HILVE II modified). | 8.2 (1.2) |  | .90 |
|  | **Others** |  |  |  |
| 14 | Independently of the lesson I just attended I find the subspecialty of internal medicine, which was addressed in that lesson, very interesting. (self) | 7.6 (1.7) | – | – |

Notes
^1^ Item 1 removed after CFA indicated low item loading on the factor
^2^ See Table 2
^3^ all reported item loadings are after item 1 has been removed except for the factor loading of item 1; factor loadings of the subscales are on the superordinate factor ‘global teaching quality’
